# Supplementary material for: The anterior paired lateral neuron normalizes odour-evoked activity in the Drosophila mushroom body calyx
Source: eLife. 2021 Dec 29;10:e74172. doi: 10.7554/eLife.74172 (PMC8741211; doi:10.7554/eLife.74172)
Supplement: Transparent reporting form [file elife-74172-transrepform1.docx]

***eLife’s* transparent reporting form**

We encourage authors to provide detailed information *within their submission* to facilitate the interpretation and replication of experiments. Authors can upload supporting documentation to indicate the use of appropriate reporting guidelines for health-related research (see [EQUATOR Network](http://www.equator-network.org/%20)), life science research (see the [BioSharing Information Resource](https://biosharing.org/)), or the [ARRIVE guidelines](http://www.plosbiology.org/article/info:doi/10.1371/journal.pbio.1000412) for reporting work involving animal research. Where applicable, authors should refer to any relevant reporting standards documents in this form.

If you have any questions, please consult our Journal Policies and/or contact us: [editorial@elifesciences.org](mailto:editorial@elifesciences.org).

**Sample-size estimation**

- You should state whether an appropriate sample size was computed when the study was being designed
- You should state the statistical method of sample size computation and any required assumptions
- If no explicit power analysis was used, you should describe how you decided what sample (replicate) size (number) to use

Please outline where this information can be found within the submission (e.g., sections or figure legends), or explain why this information doesn’t apply to your submission:

In our Material & Methods section, under Statistics, we state: “Statistical power analysis was conducted in G*Power (Faul et al. 2007)”

**Replicates**

- You should report how often each experiment was performed
- You should include a definition of biological versus technical replication
- The data obtained should be provided and sufficient information should be provided to indicate the number of independent biological and/or technical replicates
- If you encountered any outliers, you should describe how these were handled
- Criteria for exclusion/inclusion of data should be clearly stated
- High-throughput sequence data should be uploaded before submission, with a private link for reviewers provided (these are available from both GEO and ArrayExpress)

Please outline where this information can be found within the submission (e.g., sections or figure legends), or explain why this information doesn’t apply to your submission:

Information about replicates is reported per each graph in the relative figure legend as well as within the main text, when the result of a particular experiment is being described. Moreover, we write in our Material & methods section: “N values indicate number of flies hence biological replicates”

**Statistical reporting**

- Statistical analysis methods should be described and justified
- Raw data should be presented in figures whenever informative to do so (typically when N per group is less than 10)
- For each experiment, you should identify the statistical tests used, exact values of N, definitions of center, methods of multiple test correction, and dispersion and precision measures (e.g., mean, median, SD, SEM, confidence intervals; and, for the major substantive results, a measure of effect size (e.g., Pearson's r, Cohen's d)
- Report exact p-values wherever possible alongside the summary statistics and 95% confidence intervals. These should be reported for all key questions and not only when the p-value is less than 0.05.

Please outline where this information can be found within the submission (e.g., sections or figure legends), or explain why this information doesn’t apply to your submission:

Information about the statistical test used as well as p-values per each experiment can be found in the relative figure legend as well as within the main text, when the result of a particular experiment is being described. Moreover, we write in our Material & methods section, under Statistics: “Parametric (t-test, ANOVA) or non-parametric tests (Wilcoxon, Mann-Whitney, Kruskal-Wallis, Kolmogorov-Smirnov) were used depending on whether data passed the D’Agostino-Pearson normality test”. Additionally, .csv tables containing the raw data per each analysis can be found in the dedicated Dryad data repository (link available in the Manuscript).

(For large datasets, or papers with a very large number of statistical tests, you may upload a single table file with tests, Ns, etc., with reference to sections in the manuscript.)

**Group allocation**

- Indicate how samples were allocated into experimental groups (in the case of clinical studies, please specify allocation to treatment method); if randomization was used, please also state if restricted randomization was applied
- Indicate if masking was used during group allocation, data collection and/or data analysis

Please outline where this information can be found within the submission (e.g., sections or figure legends), or explain why this information doesn’t apply to your submission:

Flies carrying the genotype of interest were randomly selected from the progeny of a cross. Hence, no particular group allocation has been performed for the majority of our experiments. For the APL ON vs APL OFF experiment (Figure 4), flies were assigned to one of the two groups based on the expression or not of an mCherry tag coexpressed with the silencer tool. We explain this allocation in our Material and Methods section under “Confocal Imaging”.

**Additional data files (“source data”)**

- We encourage you to upload relevant additional data files, such as numerical data that are represented as a graph in a figure, or as a summary table
- Where provided, these should be in the most useful format, and they can be uploaded as “Source data” files linked to a main figure or table
- Include model definition files including the full list of parameters used
- Include code used for data analysis (e.g., R, MatLab)
- Avoid stating that data files are “available upon request”

Please indicate the figures or tables for which source data files have been provided:

Source data per each of the figure presented in our manuscript are available in the dedicated Dryad data repository (link available in the manuscript). In particular, in our Material & methods section, we write: “Tables containing source data per each graph can be found at: <https://doi.org/10.5061/dryad.bk3j9kdd1>“
